# Quiz Compends

**Published:** 1902-02

**Authors:** 


QUIZ COMPENDS. No. 16 A Compend of Diseases of the Skin. By JAY F. SCHAMBERG, A. B., M. D., Professor of Diseases of the Skin, Philadelphia Polyclinic and College for Graduates in Medicine Second edition, revised and enlarged. With 105 illustrations. Philadelphia: P. Blakiston's Son & Co. 1900. [Price, 80 cents.]

The second edition of this work is designed both for students and practitioners and has just been published, following the first with unusual rapidity. The number of practitioners who today are seeking instruction in compact form concerning special branches is continually increasing and there is therefore a ready market for this volume.

The author presents in a concise and accurate manner the salient principles of dermatology. There is no pretence at originality, and there are occasional inaccuracies of statement, but the work is, nevertheless, a useful one for the busy doctor.

The profuse illustrations form one of its most admirable features and will serve not only to make the subject easier to comprehend but impress the more important point of the text upon the reader. This book is one of the best in its class, and is sure to be in demand.—G.W.W.

